# Outbreak of severe acute respiratory infections caused by recombinant human adenovirus type B 7/3 in hospitalized infants from a nursery in Dakar, April 2024

**DOI:** 10.1016/j.ijregi.2024.100473

**Published:** 2024-10-16

**Authors:** Mamadou Malado Jallow, Abiboulaye Sall, Moussa Moise Diagne, Mamadou Korka Diallo, Marie Pedapa Mendy, Mamadou Aliou Barry, Alice Ingabiré Goumba, Samba Niang Sagne, Déborah Goudiaby, Cheikh Loucoubar, Ousmane Faye, Gamou Fall, Boubacar Diallo, Abdourahmane Sow, Ndongo Dia

**Affiliations:** 1 Institut Pasteur de Dakar, Département de Virologie, Dakar, Sénégal; 2 Institut Pasteur de Dakar, Direction de Santé Publique, Dakar, Sénégal; 3 Pédiatrie, Hôpital Principal de Dakar, Dakar, Sénégal; 4 Institut Pasteur de Dakar, Unité d'Epidémiologie des maladies infectieuses, Dakar, Sénégal

**Keywords:** Outbreak, Human adenovirus, Recombination, Infants, Nursery, Dakar

## Abstract

**Objectives:**

Acute respiratory infections are among the leading cause of mortality in children under 5 years of age worldwide, with most of these deaths due to bronchiolitis and pneumonia. We investigated and analyzed a pediatric outbreak of acute respiratory infections that resulted in the hospitalization of four infants in a nursery in Dakar in late April 2024.

**Methods:**

Nasopharyngeal specimens were collected from infants and tested for a panel of respiratory pathogens by multiplex real-time reverse transcription–polymerase chain reaction. Subsequently, the positive samples underwent next-generation sequencing for molecular analysis.

**Results:**

Human adenovirus (HAdV) was the principal etiologic agent detected in hospitalized infants with pneumonia and nearly half (46.7%; seven of 15) of the suspected cases identified during the investigation at the nursery. All the HAdV isolates were classified as being of subgroup B1. Molecular characterization revealed that infants attending the nursery were infected with a recombinant HAdV strain containing an adenovirus serotype 7 penton and serotype 3 hexon and fiber proteins.

**Conclusions:**

Our findings reinforce previous evidence that recombination leads to the emergence of new adenovirus strains with epidemic and lethal potential. These results emphasize the need to strengthen surveillance in inpatient settings across the country.

## Highlights

• An outbreak of severe acute pneumonia was investigated in a nursery in Dakar in April 2024.
• Human adenovirus was identified as the main causative agent in hospitalized infants.
• Human adenovirus was identified as recombinant type B 7/3 strain.
• The findings emphasize the need for enhanced monitoring in pediatric hospital settings.

## Introduction

Acute respiratory infections (ARIs) are among the leading cause of mortality in children under 5 worldwide, primarily due to bronchiolitis and pneumonia [1]. The majority of ARIs are caused by viruses, primarily rhinovirus, influenza virus, or human adenovirus (HAdV). HAdV is a non-enveloped icosahedral pathogen composed of double-stranded linear DNA belonging to the family *Adenoviridae*, genus *Mastadenovirus* [2], and was first reported as a viral pathogen in 1953 [3]. Since its identification, HAdV has been associated with a wide spectrum of diseases in human. HAdVs have been classified into seven species (HAdV-A through HAdV-G), and more than 100 genotypes have been identified. Homologous recombination, especially between the hexon, penton, and fiber genes, is the primary driver of the evolutionary trajectory and genetic diversity of HAdVs [4]. In recent years, outbreaks of severe ARI associated with recombinant HAdV-B7/3, sometimes with fatal outcomes, have been reported worldwide [5,6]. This highlights the importance of understanding its epidemiology, clinical manifestations, and risk factors. Here, we describe and analyze an outbreak of ARI caused by recombinant HAdV-B7/3 in a nursery in Dakar, which resulted in the hospitalization of four infants.

## Methodology

### Notification of cases and screening of respiratory pathogens

On April 29, 2024, the head of the pediatric department at Hôpital principal de Dakar informed the Institut Pasteur de Dakar (IPD) about four infants from the same nursery in the southern district of Dakar who were recently hospitalized for severe acute pneumonia with sudden worsening. Respiratory specimens were collected from each infant and transported to IPD for screening of respiratory pathogens within 24 hours. Specimens were screened for 26 respiratory pathogens, including 19 viruses and seven bacteria using a multiplex real-time reverse transcription–polymerase chain reaction with the Allplex Respiratory Full Panel Assay (Seegene, Seoul, Republic of Korea) as previously described [7].

### Investigation of pneumonia cases at the nursery

The severe nature of the disease prompted an epidemiological investigation at the nursery on April 30 by a joint team from the virology, microbiology, and epidemiology departments of the IPD. The nursery housed 84 infants and young children cared for by a team of nannies. For the purposes of our investigation, we defined a suspected case of ARI as any child residing at the nursery from April 1 to 30 with a body temperature over 38°C or a history of fever and at least one respiratory symptom, such as cough, dyspnea, or shortness of breath. Nasopharyngeal specimens were collected from suspected cases and analyzed using multiplex real-time reverse transcription–polymerase chain reaction, whereas asymptomatic children were closely monitored for respiratory symptoms.

### Molecular characterization

HAdV isolates were genetically characterized by using whole genome sequencing on an Illumina sequencing platform with the Twist Respiratory Virus Research Panel (103067; Twist Biosciences, San Francisco, CA, USA), as previously described [7]. The RDP4 software was used to identify potential recombination events within the HAdV genomes.

## Results

The four hospitalized infants, all males aged 3 to 6 months, with no underlying conditions, had fever up to 40.7°C, breathing difficulties, and persistent cough. The first patient had an onset on April 15 and was hospitalized on April 17. Three more infants were successively hospitalized on April 21, 25, and 27. On admission, infants were hospitalized in pediatric emergency and initially treated with broad-spectrum antibiotics combined with antipyretic and oxygen therapy. The first laboratory examinations, including complete blood count, C-reactive protein, and blood cultures, were not contributory, raising suspicion of viral respiratory infection. All four infants tested positive for HAdV and negative for other respiratory pathogens, except one infant, had a mixed infection of HAdV and *Haemophilus influenza* was encountered.

During the investigation, 15 children met the case definition, including three infants who shared the same dormitory as the hospitalized infants. The children ranged from 3 to 14 months old, with a mean age of 7 months, and 60% were female. As expected, HAdV was the most frequently detected virus, with a detection rate of 46.7% (seven of 15). Other detected viral pathogens included rhinovirus in five children (33.3%), enterovirus in two children (13.3%), and HCoV-OC43 in one child (6.7%). A triple viral infection of adenovirus/enterovirus/rhinovirus was encountered in one child. *Haemophilus Infuenzae* was found in all 15 children and *Streptococcus Pneumoniae* in 12 children. Three complete HAdV genome sequences were obtained, and the Basic Local Alignment Search Tool analysis showed the highest similarity (99.29% and 99.08%) to recombinant HAdV-B 7/3 strains detected in India in 2023 (OR130173 and OR039269).

**Figure 1:**
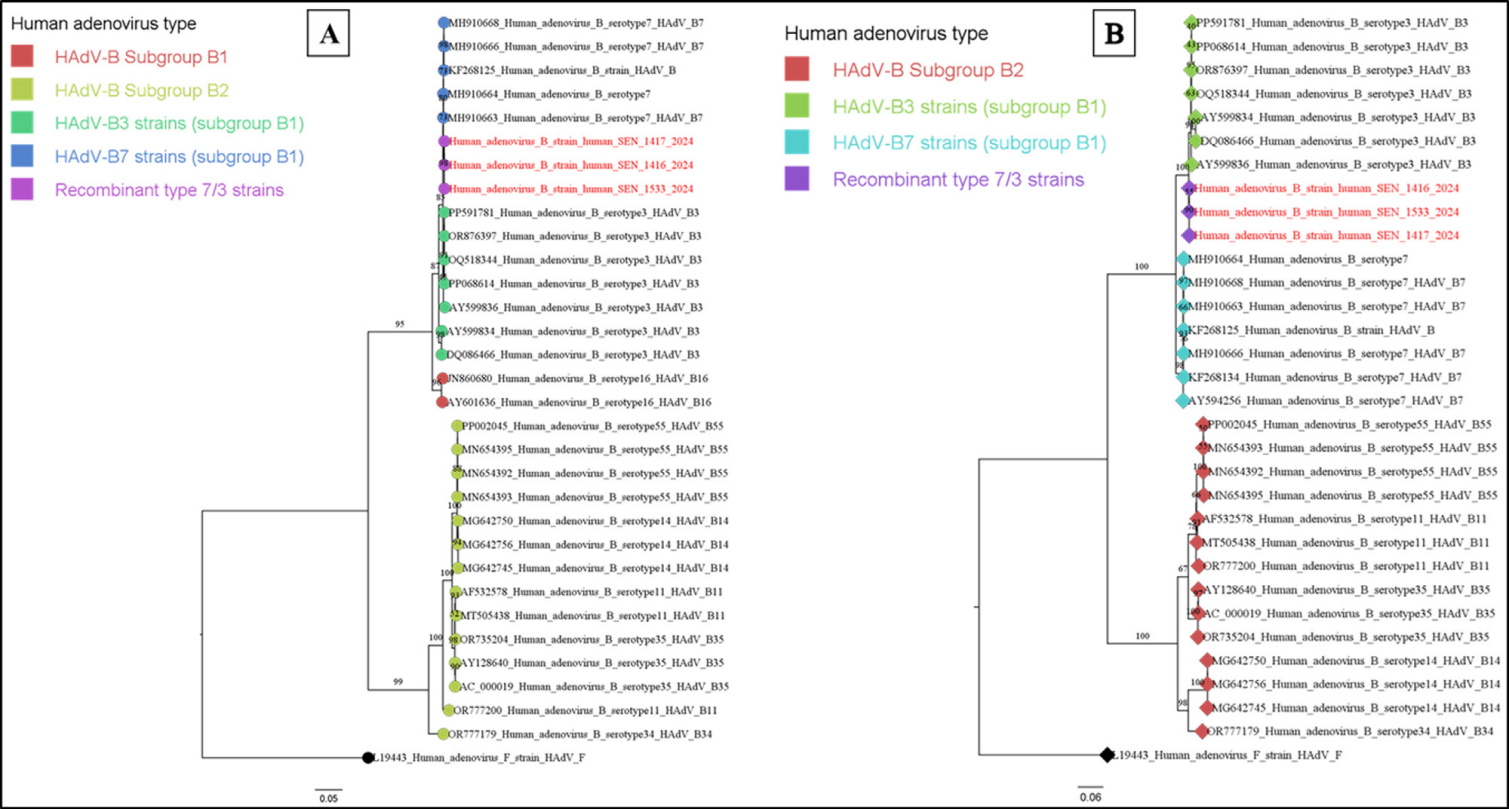
Maximum likelihood phylogenetic trees based on nucleotide sequences of the penton (a) and hexon (b) genes of HAdV-B. The trees were constructed using IQ-TREE (v.2.0.6) and visualized with Figtree software version 1.4.4. Statistical significance was tested by 1000 bootstrap replicates, and the software determined the best-fit model. Sequences highlighted in red represent recombinant HAdV-B7/3 strains associated with the outbreak at the nursery in Dakar. The trees were rooted using an HAdV type F strain. Values on the trees represent bootstrap support values, indicating the robustness of the clades in the phylogenetic trees.

The maximum likelihood phylogenetic trees based on the hexon and fiber genes showed that HAdV isolates from this outbreak clustered with serotype 3 of HAdV-B, whereas the penton gene analysis indicated they belonged to serotype 7 of HAdV-B (Figure 1).

**Figure 2:**
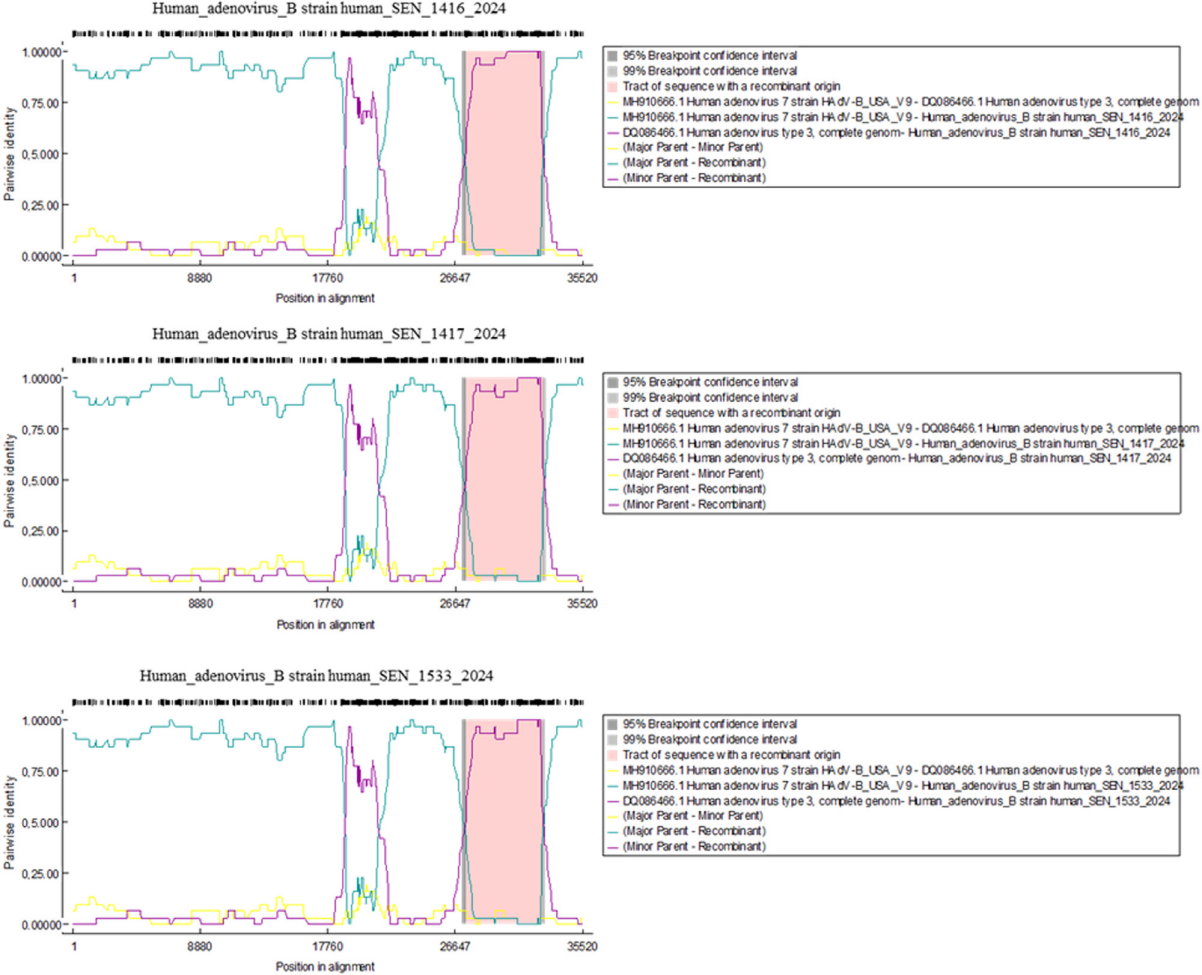
An analysis of potential recombination events within the complete genome sequences of the three Senegalese human adenovirus was conducted using the recombination detection program package Beta 4.97 in default mode. Potential major and minor parents of these recombinant strains are shown as dark cyan and purple lines, respectively, along with the sequence break points.

Using the RDP4 software, after scanning the genomes of all three HAdV strains, two potential recombination events were encountered. One recombination event, resulting from a major parent strain of HAdV-B7 (MH910668; 99.8% similarity) and a minor parent strain of HAdV-B3 (PP591781; 99.7% similarity), had a beginning breakpoint at position 18,838 (without gap) and an ending break point at position 21,524 (without gap), covering genes encoding the hexon and 23 kDa proteins. Another recombination event, resulting from major parent strain of HAdV-B7 (MH910666; 99.5% similarity) and minor parent strain of HAdV-B3 (DQ086466, 99.8% similarity), had a beginning break point at position 27,311 (without gap) and an ending break point at position 32,545 (without gap), encompassing the genes encoding the E3 and fiber protein (Figure 2).

## Discussion

In this study, we report an outbreak of HAdV infection in a nursery in Dakar, which resulted in the hospitalization of four infants in pediatric emergency, with no fatalities. Although previous reports of adenovirus outbreaks in similar environments, such as boarding schools and police and military recruit camps have resulted in fatalities [8,9], the absence of fatal cases in our study could be attributed to several factors, including early detection, timely medical intervention, and supportive care provided to the affected infants. The detection of large amounts of adenovirus DNA in the infants’ nasopharyngeal samples, along with the absence of other significant respiratory pathogens, suggests that the virus caused the ARI outbreak at the nursery. This is supported by the clinical course of infected children, which included fever, cough, dyspnea, and pneumonia, as well as epidemiological data from the outbreak that are consistent with clinical courses described in previous reports on HAdV associated ARI [6,10].

Although the virus involved in this outbreak had serotype 7 penton and serotype 3 hexon and fiber proteins, similar to strains associated with more severe outcomes elsewhere [5,6], no direct correlation between the recombinant genome and the absence of fatal cases in our study could be confirmed. This highlights the need for further investigation to clarify the role of recombinant strains in disease severity. These findings emphasize the importance of enhancing local genomic surveillance to better understand the genetic diversity of viral pathogens and to identify novel strains with epidemic and lethal potential.

The severe nature of the disease led to an epidemiological investigation at the nursery and, unsurprisingly, HAdV was the most common pathogen detected. All children with confirmed adenovirus infection also had *Haemophilus Influenzae* and *Streptococcus Pneumoniae*. Similar findings were reported during an adenovirus type 1 outbreak in a French intensive care unit [10]. The source of the infection is unknown; however, it is likely that a nanny contracted HAdV in the community and spread it to the children. These findings highlight the need to strengthen surveillance in inpatient settings across the country.

## Declarations of competing interest

The authors have no competing interest to declare.
